# Effect of packaging materials on lycopene vitamin C and water activity of dried tomato (*Lycopersicon esculentum* Mill.) powder during storage

**DOI:** 10.1002/fsn3.3562

**Published:** 2023-07-11

**Authors:** Lelise Tilahun Dufera, Werner Hofacker, Albert Esper, Oliver Hensel

**Affiliations:** ^1^ Department of Post‐Harvest Management Jimma University Jimma Ethiopia; ^2^ Institute of Applied Thermo and Fluid Dynamics, Konstanz University of Applied Sciences Konstanz Germany; ^3^ INNOTECH Mbh Stuttgart Germany; ^4^ Department of Agricultural Engineering University of Kassel Witzenhausen Germany

**Keywords:** packaging, solar drying, storage, tomato powder

## Abstract

In this work, a storage study was conducted to find suitable packaging material for tomato powder storage. Experiments were laid out in a single factor completely randomized design (CRD) to study the effect of packaging materials on lycopene, vitamin C moisture content, and water activity of tomato powder; The factor (packaging materials) has three levels (low‐density polyethylene bag, polypropylene bottle, wrapped with aluminum foils, and packed in low‐density polyethylene bag) and is replicated three times. During the study, a twin layer solar tunnel dried tomato slices of var. Galilea was used. The dried tomato slices were then ground and packed (40 g each) in the packaging materials and stored at room temperature. Samples were drawn from the packages at 2‐month interval for quality analysis and SAS (version 9.2) software was used for statistical analysis. From the result, higher retention of lycopene (80.13%) and vitamin C (49.32%) and a nonsignificant increase in moisture content and water activity were observed for tomato powder packed in polypropylene bottles after 6 months of storage. For low‐density polyethylene packed samples and samples wrapped with aluminum foil and packed in a low‐density polyethylene bag, 57.06% and 60.45% lycopene retention and 42.9% and 49.23% Vitamin C retention were observed, respectively, after 6 months of storage. Considering the results found, it can be concluded that lycopene and vitamin C content of twin layer solar tunnel dried tomato powder can be preserved at ambient temperature storage by packing in a polypropylene bottle with a safe range of moisture content and water activity levels for 6 months.

## Graphical abstract

Polypropylene bottle packaging retained 80.13% of lycopene and 9.32% of vitamin C in tomato powder stored for 6 months. Tomato powder can be packed in polypropylene bottle for better quality at ambient temperature storage.

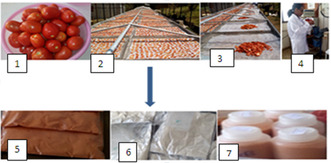

## 1 INTRODUCTION

These days, tomato (*Lycopersicon esculentum* Mill.) is becoming the most popular crop and a desirable functional ingredient in foods as a result of its significant content of vitamins and a wide variety of phytochemicals and its reported health benefits (Stratakos et al., 2016). It has the strongest evidence for its protection against stomach prostate and lung cancer (Diener & Christian, 2008). Vitamin C and lycopene are the predominant compounds found in tomato that exhibit the highest antioxidant activity (Stratakos et al., 2016). However, tomato fruit has short storage life due to its high moisture content and deteriorates very fast after harvest (Kaur et al., 2020) leading to high postharvest losses. Moreover, tomato is a seasonal crop, and when there is seasonal glut, large percentage of it will be lost by improper postharvest handling and processing. In Ethiopia, about 25%–40% of postharvest loss of tomato is reported by different researchers during different postharvest handling stages (Abera et al., 2020; Asrat et al., 2019; Emana et al., 2017).

Of all food preservation methods, drying is the one that is most commonly applied to preserve tomatoes (Qiu et al., 2019). Worldwide several studies have addressed the effect of drying temperatures and methods and different predrying treatments on tomato quality. Different researchers assessed the influence of predrying treatments (Farooq et al., 2020; Hameed et al., 2016; Mwende et al., 2018) and the effect of drying temperatures (Azeez et al., 2019; Lutz et al., 2015; Muratore et al., 2008; Mwende et al., 2018; Yusufe et al., 2017) on quality of tomato but few have focused on packaging materials to be used during storage.

Packaging materials are one of the last steps in food industries to extend the conservation of dried fruits (Udomkun et al., 2016). Storage of tomato powder may result in quality loss depending on the storage condition (water activity of the product, oxygen and light permeability of the packaging and storage temperature) (Martínez‐Hernández et al., 2016). In tomato processing, a good drying method must be followed by a good packaging method to maintain the quality parameters during storage.

In our previous work of drying tomato slices in twin layer solar tunnel (Dufera et al., 2021b), a promising result was obtained in quality retention. The obtained result in this work is comparable with the result reported for energy‐intensive mechanical drying methods.

In addition to minimizing the chemical degradation reactions during processing, maximizing the conservation of important nutrients during storage is also a challenge in modern food sciences (Udomkun et al., 2016). Previous studies done on tomato preservation in Ethiopia were mainly focused on the effect of drying conditions on dried tomato quality (Dufera et al., 2021b; Hrabe & Herak, 2015; Yusufe et al., 2017). However, a comprehensive study on finding packaging materials suitable for tomato powder storage to be used by the producers and consumers in Ethiopia was not presented. Therefore, in this study, efforts were made to find out suitable packaging material for twin layer solar tunnel dried tomato powder storage based on its lycopene, vitamin C, and water activity changes during storage of 6 months.

## 2 MATERIALS AND METHODS

### 2.1 Description of the study site

This research was carried out at Jimma University College of Agriculture and Veterinary Medicine located in Jimma zone, Ethiopia at about 346 km from Addis Ababa in southwest and lies between 36°5′ E longitude and 7°42′ N latitude and at an altitude of 1710 m above sea level (masl).

### 2.2 Experimental materials and sample preparation

**FIGURE 1:**
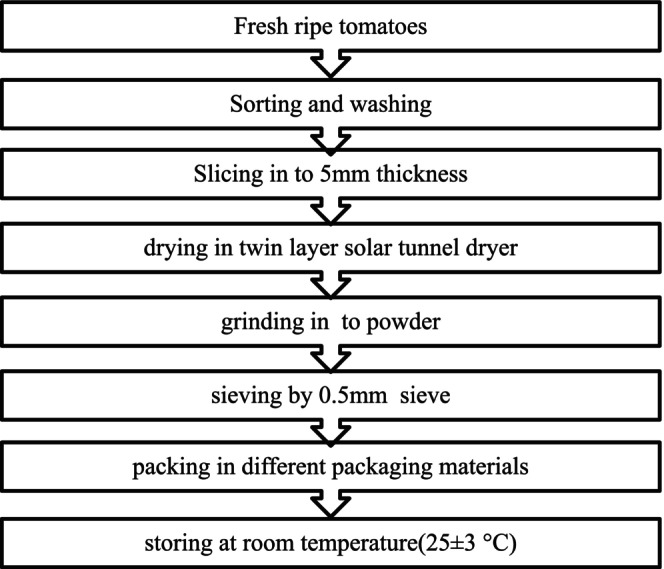
Process flow  chart of tomato powder preparation and storage.

**FIGURE 2:**
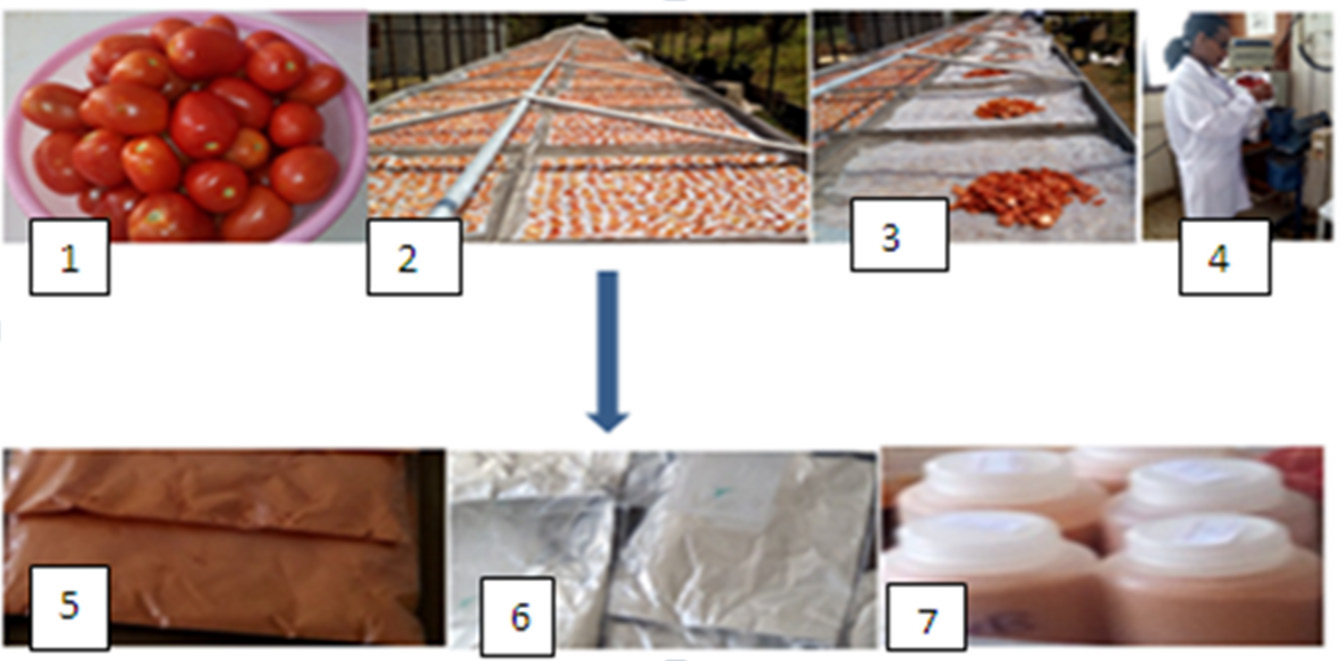
Tomato powder preparation and storage. 1 = Fresh tomato, 2 = Tomato slices being dried in solar tunnel dryer, 3 = Dried tomato slices, 4 = Grinding tomato slices with laboratory grinder, 5 = Tomato powder packed in polyethylene bag, 6 = Tomato powder packed in polyethylene bag by wrapping with aluminum foil, 7 = Tomato powder packed in polypropylene bottle.

The drying experiment was conducted with 180 kg of fresh tomatoes var. Galilea (3 kg of tomato slices per tray). The tomatoes were hand harvested from the field of a local farmer in Mojo and transported to Jimma. The fully mature tomatoes were sorted, washed, sliced into 5 mm thickness, and put into the twin layer solar tunnel drier to dry (Figures 1 and 2). During drying, Testo, model 174, Germany, was placed at 4‐m interval inside the drier to measure the temperature and relative humidity. Maximum drying air temperature recorded were 53.3°C, 58.2°C, and 61.2°C at collector outlet (0 m), middle of the dryer (8 m), and at dryer outlet (16 m), respectively, and 38.2°C for ambient air. The minimum relative humidity values were 7.4%, 6.2%, and 7.7% at collector outlet, middle of the dryer, and at dryer outlet, respectively, and 14.3% for ambient air. Sun drying was used as control. Quality retention observed in the dried tomatoes with respect to the quality of fresh tomatoes and details regarding the kinetics of drying has been presented and discussed in our previous papers (Dufera et al., 2021a, 2021b). Then, the same sample dried in the above two articles was used in storage study.

### 2.3 Storage study

The dried tomato slices were ground into powder using laboratory grinder of model Karl Kolb D‐6072 Germany and sieved by 0.5‐mm sieve to produce a fine powder. The packaging materials are low‐density polyethylene (LDPE) bag, aluminum foil (the aluminum foil was used only for wrapping the samples to protect from exposure to light but not sealed for protection of oxygen and moisture), i.e., the sample is wrapped with aluminum foil and packed in polyethylene bag. Therefore, the oxygen and water vapor permeability are expected to be better than polyethylene but not equivalent to aluminum foil, and polypropylene bottles (PP) were purchased from a local supermarket and used in the study (Figure 2). These packaging materials were selected to be used for this experiment considering their availability in the market with affordable price and they are widely used in the storage and handling of different flours in Ethiopia (Table 1). The samples were packed 40 g per each packaging material and replicated nine times for each packaging. Then, all the samples were stored at 25 ± 3°C (room temperature) for 6 months. At 2‐month interval, samples were then drawn from nine of the pouches (three for each packaging) and their contents were analyzed for vitamin C, lycopene, water activity, and moisture. Due to facility limitation to control relative humidity, it is tried to manage moisture migration to the samples by drawing samples only once from each packaging material throughout the sampling period.

**TABLE 1:** Water vapor and oxygen permeability of the evaluated packaging materials.

Packaging material	Thickness (μm) (measured)	OP (cm^3^/m^2^d bar)	WVP (g/m^2^ d)	Reference
Polyethylene	50	50–200	0.5–2	(Lange & Wyser, 2003)
Polypropylene	1000	50–100	0.2–0.4	(Lange & Wyser, 2003)
Aluminum foil	20	<0.05	<0.05	(Amberg‐Schwab, 2005)

### 2.4 Quality parameters

#### 2.4.1 Lycopene

Lycopene content of tomato samples was determined as described in (Suwanaruang 2016) by extracting with ethanol: acetone: hexane (1:1:2) (v:v:v) mixture. Powdered tomato sample (0.1 g) was dissolved in 1‐mL distilled water and vortexed in water bath for 1 h at 30°C. Then, 8.0 mL of acetone, ethanol, and hexane, (ratio 1:1:2) was added, capped, and vortexed again, followed by incubation in a dark place for 60 min. Then, one milliliter of distilled water was added to the samples and vortexed and allowed to stand and separate into phases. Ultraviolet–Visible spectrophotometer (T80, China) was used to measure the absorbance of the upper layers of the lycopene samples at wavelength of 503 nm. Lycopene content (mg/100 g dw) of the samples was then calculated using Equation 1

(1) $$\text{Lycopene}\;\left(\frac{\mathrm{mg}}{100\;g\;\mathrm{dw}}\right)={\mathrm{Abs}}_{503\;\mathrm{nm}}\times 537\times 8\times \frac{0.55}{0.1}\times 172$$

where Abs_503 nm_ is the absorbance at 503 nm; 537 = lycopene molecular weight in g/mole; 8 = the mixed solvent volume in milliliter; 0.55 = the volume ratio of the upper layer to the mixed solvents in milliliter; 0.10 = the weight of tomato added in gram; 172 = the extinction coefficient for lycopene in hexane in mM^−1^ (Anthon & Barrett, 2006).

#### 2.4.2 Vitamin C

Titration by 2,6‐dichloroindophenol was used to determine vitamin C according to the AOAC (2005). Briefly, 0.2 g of dried tomato powder was mixed in 40 mL of HPO_3_‐HOAc extracting solution (i.e., 40 mL of HOAc (acetic acid) and 15 g of HPO_3_ (metaphosphoric acid) in 500 mL of deionized H_2_O). A quantity of 50 mg of L‐ascorbic acid was diluted in 50 mL of HPO_3_‐HOAc extracting solution and diluted to a final concentration of 10 mg of ascorbic acid/100 mL to prepare the standard solution. Then, 10 mL of standard solution, test sample, and blank solution were titrated with the indophenol reagent (i.e., prepared by dissolving 50 mg of 2,6‐dichloroindophenol sodium salt and 42 mg of NaHCO_3_ to 200 mL with deionized H_2_O) to a light but distinctive rose pink endpoint lasting *≥*5 s. Vitamin C content of the sample in mg/g was then calculated using:

$$\text{Vitamin}\;C=\left(A-B\right)\times \left(C/D\right)\times \left(V/Y\right)$$

where *A* = average volume (ml) of test solution titration, *B* = average volume (ml) of test blank titration, C = mass (mg) of ascorbic acid equivalents to 1.0‐ml indophenol standard solution, *D* = sample weight (g), and *V* = volume of initial test solution (ml) and *Y* = volume of test solution titrated (ml).

#### 2.4.3 Water activity

The water activity of fresh and dried tomato slices was measured by water activity meter (model Novasina AG, CH‐8853 Lachen) at room temperature (23.4 ± 1°C).

#### 2.4.4 Moisture content

Moisture content of the samples was determined by air oven (Leicester, LE67 5FT, England) dying method according to AOAC (2011).

### 2.5 Experimental design

Completely randomized design (CRD) was used for the comparative evaluation of the effect of packaging materials on quality of tomato slices. The factor (packaging material) has three levels (low‐density polyethylene bag, polypropylene bottle, and wrapped with aluminum foil and packed in polyethylene bag) with three replications.

### 2.6 Statistical analysis

Statistical analysis was performed over the storage periods. Analyses of variance (ANOVA) were done for each quality parameter based on the procedures described by Gomez and Gomez (1984). SAS Version 9.2 was used for ANOVA in CRD with three replications. For parameters having significant mean differences, the treatment means were compared using Tukey's test at 5% probability level.

## 3 RESULTS

### 3.1 Quality of stored tomato powder

Packaging materials showed a statistically significant difference in the lycopene content of stored tomato powder (Table. 2). Higher retention of lycopene was observed in polypropylene bottle packed tomato powder (80.13%) followed by samples wrapped with aluminum foil and packed in low‐density polyethylene bag (60.45%). The lowest retention was observed in low‐density polyethylene bag packed tomato powder (Table. 2).This possibility of entrance of oxygen and water vapor in aluminum foil wrapped and packed in low‐density polyethylene bag samples has resulted in higher increase in water activity of the tomato powder and lower retention of lycopene than the samples packed in polypropylene bottle.

**TABLE 2:** Effect of packaging material on lycopene and vitamin C content of tomato powder.

Packaging	Months of storage	Lycopene (mg/100 gDM)	Vitamin C (mg/100 gDM)
Polypropylene bottle	0	102.5 ± 0.057	50.57 ± 0.025
	2	82.4^a^ ± 1.94	48.54^a^ ± 4.85
	4	82.643^a^ ± 2.58	39.80^a^ ± 3.4
	6	82.138^a^ ± 1.54	24.94^a^ ± 1.12
Low‐density polyethylene bag	0	102.5 ± 0.057	50.57 ± 0.025
	2	72.925^b^ ± 0.18	38.83^b^ ± 0.00
	4	71.036^b^ ± 1.64	31.84^b^ ± 3.4
	6	58.487^b^ ± 5.25	17.47^c^ ± 0.00
Aluminum foil in low‐density polyethylene bag	0	102.5 ± 0.057	50.57 ± 0.025
	2	80.79^a^ ± 1.98	45.30^ab^ ± 4.39
	4	78.471^ab^ ± 5.49	35.82^ab^ ± 3.44
	6	61.967^b^ ± 1.09	21.71^b^ ± 0.00

*Note*: Means sharing the same letter along the same column are not significantly different (*p* ≥ .05) according to Tukey's (significant difference) test.

The lycopene retention obtained in this study (80.13%–57.06%) is much higher than the retention obtained by using high‐density polyethylene (7.9%), medium‐density polyethylene (6.6%), and laminated aluminum foil (12.48%) which is reported by Sarker et al. (2014) during studying the storage of tomato powder in different packaging materials for 6 months.

Retention of vitamin C is usually used as indicator for nutrient retention in all food products; it is of great importance to investigate its residual level in tomato powder (Obadina et al., 2018). In this study, vitamin C content of the samples packed in all packaging used was decreased significantly during storage. A statistically significant difference was observed in the vitamin C content of tomato powder packed in different packaging materials after storage. The highest vitamin C retention (49.32%) was recorded in polypropylene bottle packed tomato powder followed by samples wrapped with aluminum foil and packed in low‐density polyethylene bag (42.93%) after 6 months of storage (Table 2).

In this study, a significant difference was obtained in water activity value of the stored tomato samples (Table 3). The water activity values of the samples were increased from 0.33 to 0.432, from 0.33 to 0.55, and from 0.33 to 0.54 for samples packed in polypropylene bottle, low‐density polyethylene bag, and samples wrapped in aluminum foil and packed in low‐density polyethylene bag, respectively.

**TABLE 3:** Effect of packaging material on water activity (Wa) and moisture content of tomato powder.

Packaging	Months of storage	Water activity	Moisture content (%)
Polypropylene bottle	0	0.33 ± 0.01	10.85 ± 0.02
	2	0.36^c^ ± 0.00057	11.32^b^ ± 0.43
	4	0.40^c^ ± 0.004	11.11^b^ ± 0.43
	6	0.43^c^ ± 0.00057	11.5^b^ ± 0.23
Low‐density polyethylene bag	0	0.33 ± 0.01	10.85 ± 0.02
	2	0.41^b^ ± 0.00057	12.75^a^ ± 0.1
	4	0.49^a^ ± 0.0011	12.72^a^ ± 0.56
	6	0.55^a^ ± 0.00057	15.83^a^ ± 0.17
Aluminum foil in low‐density polyethylene bag	0	0.33 ± 0.01	10.85 ± 0.02
	2	0.42^a^ ± 0.00057	12.53^a^ ± 0.08
	4	0.47^b^ ± 0.0005	12.43^a^ ± 0.00
	6	0.54^b^ ± 0.00057	14.79^a^ ± 0.64

*Note*: Means sharing the same letter along the same column are not significantly different (*p* ≥ .05) according to Tukey's (significant difference) test.

A significant increase in moisture content of tomato powder was observed in samples packed in low‐density polyethylene bag, wrapped in aluminum foil, and packed in low‐density polyethylene bag during storage. Nonsignificant increase was observed in the moisture content of tomato powder stored in the polypropylene bottle (Table 3). The moisture content of tomato powder packed in low‐density polyethylene bag was increased from 11.85% to 15.85% and after 6 months of storage and the moisture content of samples wrapped with aluminum foil and packed in low‐density polyethylene bag was increased to 14.79%.

## 4 DISCUSSION

### 4.1 Quality of stored tomato powder

The most important cause of lycopene degradation in tomato powder during storage is availability of oxygen and it is reported that low oxygen content, low moisture content, low storage temperature, and low water activity have a limiting effect on the oxidation of lycopene (Shi & Maguer, 2000). Packaging materials have different oxygen and water vapor permeability (Forsido et al., 2021) and tomato powder should be stored in moisture‐proof packaging material due to its hygroscopic nature, especially in higher RH conditions (Davoodi et al., 2007). In this study, the obtained high lycopene retention for polypropylene bottle packed tomato powder may be due to the lower oxygen and water vapor permeability of polypropylene bottle than polyethylene bag. The result obtained in this work is in agreement with the findings revealed by other researchers that water activity of the product mainly affects the stability of carotenoids. Kaur et al. (2020) reported higher decrease in lycopene contents of tomato powder after 180 days of refrigerated storage for samples having higher initial water activity. Lavelli et al. (2007) reported maximum carotenoid stability in stored tomato powder having water activity in the range of 0.341–0.537 and decreased carotenoid stability above these values.

The lycopene retention obtained in this study (80.13%–57.06%) is much higher than the retention obtained by using high‐density polyethylene (7.9%), medium‐density polyethylene (6.6%), and laminated aluminum foil (12.48%) which is reported by Sarker et al. (2014) during studying the storage of tomato powder in different packaging materials for 6 months. On the other hand, the lycopene retention obtained in this study by using polypropylene bottle (80.13%) is smaller than the retention reported by Liu et al. (2010) in using aluminum foil bag packaging after storing in the dark at 25°C and 37°C for 5 months (96.5% and 93%), respectively.

Vitamin C is highly sensitive during the storage of any food product (Kaur et al., 2020). Vitamin C retention during the storage of food materials is affected by increase in water activity moisture content and storage temperature (Udomkun et al., 2016). The decrease in vitamin C content of tomato powder observed in this study may be due to the high sensitivity of vitamin C to oxidation during storage. In this study, the observed lower vitamin C retention in polyethylene bag stored tomato powder may be due to a significant increase in water activity during storage as a result of the high water vapor permeability of polyethylene bag. These results were in agreement with the studies of Kaur et al. (2020) who reported significant loss of vitamin C contents in tomato powder after 180 days of refrigerated storage for samples having higher initial water activity. Sablani et al. (2007) also reported reduced rate of vitamin C degradation at reduced level of water activity during evaluating stability of vitamin C in fortified formula stored at room temperature at different water activities. In addition, oxygen permeability of packaging materials can promote oxidation reactions and loss of vitamins (Bauer et al., 2022). In this study, the high loss observed in vitamin content of tomato powder packed in polyethylene bag may be due to its oxygen permeability relative to polypropylene bottle.

The vitamin C retention obtained in this study (34.5%–49.32%) is much higher than the retention reported by Sarker et al. (2014) using high‐density polyethylene (8.68%), medium‐density polyethylene (5.58%), and laminated aluminum foil (32.79%) during studying the storage of tomato powder in different packaging materials for 6 months. On the other hand, the vitamin C retention obtained in this study by using polypropylene bottle (49.32%) is smaller than the retention reported by Liu et al. (2010) in using aluminum foil bag packaging (63%) after storing in the dark at 25°C for 5 months. However, the vitamin C retention result obtained in samples in this study is much higher than the retention obtained by Liu et al. (2010) in using aluminum foil bag for tomato powder storage in the dark at 37°C (4.68%) for 5 months.

The increase in water activity value of samples packed in low‐density polyethylene bag and wrapped with aluminum foil and packed in low‐density polyethylene bag in this study may be due to increase in moisture of the samples as a result of the high water vapor permeability of the polyethylene. Polypropylene has a higher barrier to water vapor than polyethylene bag (Shin & Selke, 2014). Foods having water activity values between 0.5 and 0.8 are more affected by nonenzymatic browning and values under 0.2 results in lipid oxidation (Guiné & Barroca, 2017). Water activity value of tomato powder packed in polypropylene bottle was within the safe range up to 6 months of storage.

The observed increase in moisture of samples packed in polyethylene bag is similar to the report of former studies. For tomato powder packed in polyethylene bag, Narsing Rao et al. (2011) reported an increase of moisture content from 12.29% to 18.03% moisture after 6 months of storage at room temperature. The relatively lower moisture content observed in samples packed in polypropylene bottle may be due to their lower water vapor permeability. The increase in moisture content of the aluminum foil wrapped and packed in low‐density polyethylene bag samples may be due to the fact that the aluminum foil is only used in wrapping the samples to protect light entrance from the samples and was not sealed to protect the entrance of oxygen and water vapor. The result obtained for moisture content is similar to water activity. This similarity could be due to vapor transmission through packaging materials (Forsido et al., 2021). Surface area of the packaging material is one of the factors affecting its water vapor permeability (Jaya & Das, 2005). Hence, in addition to the nature of the packaging used the difference in surface area of the packages may also contribute to the increase of moisture of the samples packed in the packaging materials.

## 5 CONCLUSIONS AND RECOMMENDATIONS

This study demonstrated that polypropylene bottle packaging of tomato powder showed the best result in retaining the highest lycopene and vitamin C content than the other packaging used. Dried tomato samples packed in polyethylene bags with or without wrapping with aluminum foil resulted in increase in water activity and moisture content during storage. Packaging material is the most important factor that significantly affected the final water activity of tomato powder. Greater retention in lycopene content of tomato powder packed in polypropylene bottle could be associated with low water activity, which makes the lycopene and vitamin C oxidation difficult. Even though vitamin C is highly sensitive to high temperature; and refrigerated storage is recommended; it is not economically affordable to use refrigerated storage in developing countries. Therefore, moisture and waterproof packaging are recommended to be used in packaging of tomato powder during storage. Generally, this finding is helpful to minimize postharvest loss of tomato powder during ambient storage to make tomato powder available with retained nutrients throughout the year.

## AUTHOR CONTRIBUTIONS

**Lelise Tilahun Dufera:** Conceptualization (equal); data curation (equal); formal analysis (lead); investigation (equal); methodology (equal); writing – original draft (lead); writing – review and editing (equal). **Werner Hofacker:** Conceptualization (equal); data curation (equal); methodology (equal); supervision (equal); writing – review and editing (equal). **Albert Esper:** Conceptualization (equal); supervision (equal); writing – review and editing (equal). **Oliver Hensel:** Conceptualization (equal); methodology (equal); project administration (equal); supervision (equal); writing – review and editing (equal).

## FUNDING INFORMATION

This work is funded by RELOAD (Reduction of postharvest losses and value addition in East African food value chain) (grant number BMBF/BMZ 031A247A‐D).

## CONFLICT OF INTEREST STATEMENT

The authors have no conflict of interest to declare.

## Data Availability

Corresponding author can provide the data used to report these results upon request.
